# Insulin Resistance Is Not Sustained Following Denervation in Glycolytic Skeletal Muscle

**DOI:** 10.3390/ijms22094913

**Published:** 2021-05-06

**Authors:** Shawna L. McMillin, Erin C. Stanley, Luke A. Weyrauch, Jeffrey J. Brault, Barbara B. Kahn, Carol A. Witczak

**Affiliations:** 1 Department of Kinesiology, East Carolina University, Greenville, NC 27858, USA; mcmil318@umn.edu (S.L.M.); erincstanley16@gmail.com (E.C.S.); weyrauchl20@ecu.edu (L.A.W.); jebrault@iu.edu (J.J.B.); 2 Department of Biochemistry & Molecular Biology, Brody School of Medicine, East Carolina University, Greenville, NC 27834, USA; 3 Department of Physiology, Brody School of Medicine, East Carolina University, Greenville, NC 27834, USA; 4 East Carolina Diabetes & Obesity Institute, East Carolina University, Greenville, NC 27834, USA; 5 Department of Anatomy, Cell Biology & Physiology, Indiana University School of Medicine, Indianapolis, IN 46202, USA; 6 Indiana Center for Musculoskeletal Health, Indiana University School of Medicine, Indianapolis, IN 46202, USA; 7 Division of Endocrinology, Diabetes & Metabolism, Beth Israel Deaconess Medical Center and Harvard Medical School, Boston, MA 02215, USA; bkahn@bidmc.harvard.edu; 8 Center for Diabetes and Metabolic Diseases, Indiana University School of Medicine, Indianapolis, IN 46202, USA

**Keywords:** fiber type, glucose transporter, insulin signaling, myosin heavy chain, type 2 diabetes

## Abstract

Denervation rapidly induces insulin resistance (i.e., impairments in insulin-stimulated glucose uptake and signaling proteins) in skeletal muscle. Surprisingly, whether this metabolic derangement is long-lasting is presently not clear. The main goal of this study was to determine if insulin resistance is sustained in both oxidative soleus and glycolytic extensor digitorum longus (EDL) muscles following long-term (28 days) denervation. Mouse hindlimb muscles were denervated via unilateral sciatic nerve resection. Both soleus and EDL muscles atrophied ~40%. Strikingly, while denervation impaired submaximal insulin-stimulated [^3^H]-2-deoxyglucose uptake ~30% in the soleus, it enhanced submaximal (~120%) and maximal (~160%) insulin-stimulated glucose uptake in the EDL. To assess possible mechanism(s), immunoblots were performed. Denervation did not consistently alter insulin signaling (e.g., p-Akt (Thr308):Akt; p-TBC1D1 [phospho-Akt substrate (PAS)]:TBC1D1; or p-TBC1D4 (PAS):TBC1D4) in either muscle. However, denervation decreased glucose transporter 4 (GLUT4) levels ~65% in the soleus but increased them ~90% in the EDL. To assess the contribution of GLUT4 to the enhanced EDL muscle glucose uptake, muscle-specific GLUT4 knockout mice were examined. Loss of GLUT4 prevented the denervation-induced increase in insulin-stimulated glucose uptake. In conclusion, the denervation results sustained insulin resistance in the soleus but enhanced insulin sensitivity in the EDL due to increased GLUT4 protein levels.

## 1. Introduction

Denervation rapidly induces insulin resistance in both oxidative and glycolytic skeletal muscles. This has been shown in numerous studies that demonstrated impairments in insulin-stimulated skeletal muscle glucose uptake 1–3 days after denervation [1,2,3,4,5,6,7,8,9,10,11,12,13,14,15]. However, whether denervation leads to sustained or permanent insulin resistance in both oxidative and glycolytic skeletal muscles is surprisingly less clear. Studies performed in rat mixed gastrocnemius muscle 35–70 days post-denervation, as well as those performed in human mixed vastus lateralis muscle strips 3–24 years post-spinal cord injury, demonstrated no change in insulin-stimulated muscle glucose uptake [16,17]. In contrast, work performed in oxidative rat soleus muscle showed an ~45% decrease in insulin-stimulated glucose uptake 28 days post-denervation [18], whereas work in glycolytic mouse extensor digitorum longus (EDL) muscle demonstrated a robust 150–200% increase in glucose uptake 28 and 56 days post-denervation [19]. Collectively, these findings may suggest that denervation leads to a sustained impairment in glucose uptake in oxidative muscle fibers, but not glycolytic muscle fibers. However, this interpretation should be considered with caution since these studies were not performed in denervated oxidative and glycolytic muscles isolated from the same animals or even species of animals. Therefore, the first goal of this study was to determine whether insulin resistance is sustained following long-term (28 days) denervation in both oxidative soleus and glycolytic EDL skeletal muscles isolated from mice.

The rapid impairment in insulin-stimulated glucose uptake following 1–3 days of denervation has been associated with changes in several key glucometabolic proteins within skeletal muscle. These changes include: (1) decreases in the insulin-stimulated phosphorylation/activation of the serine/threonine kinase, Akt [15,20]; (2) increases in the expression of glucose transporter 1 (GLUT1) [9,12,13,21,22]; (3) decreases in the expression of the insulin responsive glucose transporter 4 (GLUT4) [8,9,10,11,12,13,21,22,23,24]. Strikingly, at 28 and 56 days post-denervation, work in glycolytic mouse EDL muscle demonstrated an increase in the basal phosphorylation of Akt, as well as increases in the protein levels of both GLUT1 and GLUT4 [19], suggesting a reversal of the decrements observed in Akt signaling and GLUT4 expression shortly after denervation. Whether there is reversal in the insulin-stimulated phosphorylation of Akt signaling and/or expression of other glucometabolic proteins in either oxidative or glycolytic muscles is presently unknown. Therefore, the second goal of this study was to determine whether the changes in glucometabolic proteins observed rapidly following denervation are sustained or reversed in both oxidative soleus and glycolytic EDL mouse muscles following long-term (28 days) denervation.

## 2. Results

### 2.1. Effects of Long-Term Denervation on Skeletal Muscle Insulin Sensitivity

**Figure 1:**
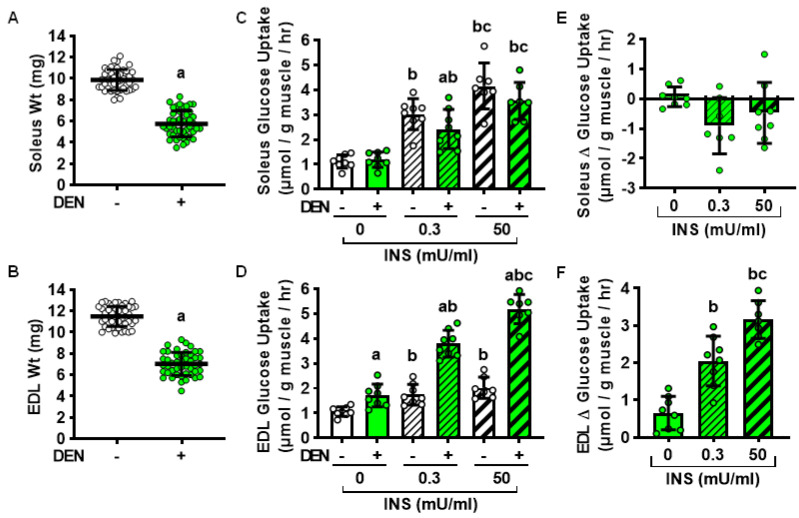
Long-term denervation (DEN) elicits sustained impairments in insulin sensitivity in soleus but not extensor digitorum longus (EDL) muscles. In male wild-type mice, hindlimb muscle DEN was induced in one leg for 28 days while the contralateral leg was sham-operated (-). (**A**) Soleus and (**B**) EDL muscle weights. (**C**,**D**) Ex vivo muscle [^3^H]-2-deoxy-d-glucose uptake was assessed in the presence of 0, 0.3 or 50 mU/ml insulin (INS). (**E**,**F**) DEN-induced change (Δ) in muscle glucose uptake relative to the contralateral control muscle. Statistical significance was defined as *p* < 0.05 and determined using paired *t*-tests (Panels (**A**,**B**); *n* = 44–46 muscles/group), two-way ANOVAs and Tukey’s post-hoc analysis (**C**,**D**; *n* = 7–8 muscles/group), or one-way ANOVAs and Tukey’s post-hoc analysis (**E**,**F**; *n* = 7–8 muscles/group). Significance is denoted by ‘a’ vs. -, ‘b’ vs. 0 mU/mL INS, and ‘c’ vs. 0.3 mU/mL INS.

To determine whether denervation induces a sustained, long-lasting impairment in both oxidative and glycolytic muscle insulin sensitivity, wild-type mice underwent unilateral resection of a segment of the sciatic nerve to induce hindlimb muscle denervation. The contralateral leg was sham-operated and served as the control. After 28 days, oxidative soleus and glycolytic extensor digitorum longus (EDL) muscles were isolated and weighed. As shown in Figure 1, long-term denervation decreased both soleus and EDL muscle weights ~40% compared to their sham controls (Figure 1A,B). Immediately after weighing, all muscles from within a single mouse were incubated in either 0, 0.3 or 50 mU/ml insulin, and [^3^H]-2-deoxyglucose uptake was assessed. In the soleus, denervation did not affect basal or maximal insulin-stimulated glucose uptake; however, it did impair submaximal insulin-stimulated glucose uptake ~30% compared to the contralateral control (Figure 1C,E). In contrast, in the EDL, denervation enhanced glucose uptake in the absence of insulin (~60%), as well as in response to both submaximal (~120%) and maximal insulin (~160%) stimulation compared to their contralateral controls (Figure 1D,F). Thus, these results demonstrate that denervation induces a sustained impairment in insulin sensitivity in the oxidative soleus but not the glycolytic EDL muscle. 

### 2.2. Long-Term Denervation and Muscle Fiber Type

**Figure 2:**
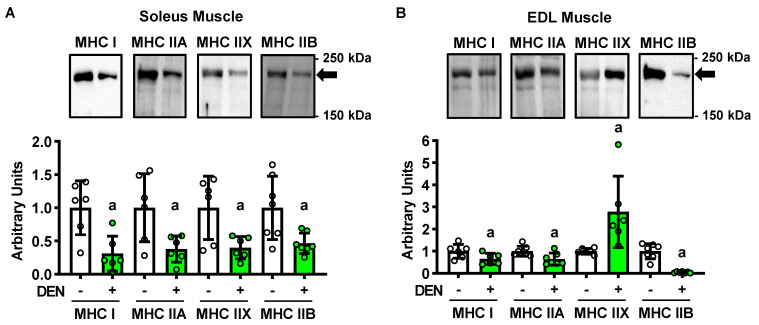
Long-term denervation (DEN) differentially alters myosin heavy chain (MHC) isoform protein levels between the soleus and extensor digitorum longus (EDL) muscles. In male wild-type mice, hindlimb muscle DEN was induced in one leg for 28 days while the contralateral leg was sham-operated (-). Muscles were excised and MHC isoform content assessed by immunoblot in the (**A**) soleus and (**B**) EDL muscles. Statistical significance was defined as *p* < 0.05, assessed by paired *t*-tests, and denoted by ‘a’ vs. -. *n* = 6–7 muscles/group.

Myosin heavy chain (MHC) isoform expression is a key indicator of muscle fiber type. Importantly, previous work has demonstrated that, while innervated mouse soleus muscles are predominantly comprised of fibers possessing, the highest rates of insulin-stimulated glucose uptake (i.e., ~80% MHC type I and IIA fibers [25,26,27]), mouse EDL muscles are largely comprised of fibers possessing the lowest rates of insulin-stimulated glucose uptake (i.e., ~56% MHC type IIB fibers) [25,27]. To determine whether the long-term denervation-induced changes in muscle insulin sensitivity were due to a switch in fiber type, MHC isoform immunoblots were performed. In the soleus, denervation decreased the levels of all MHC isoforms 40–55% (Figure 2A). In the EDL, denervation decreased MHC type I levels ~35%, IIA levels ~35%, and IIB ~95% levels, but increased MHC type IIX levels ~180% (Figure 2B). Since MHC type IIX fibers exhibit only moderate insulin-stimulated glucose uptake rates [25], collectively these findings suggest that a change in muscle fiber type is not responsible for the long-term denervation-induced changes in muscle insulin sensitivity.

### 2.3. Long-Term Denervation and the Insulin-Signaling Cascade

**Figure 3:**
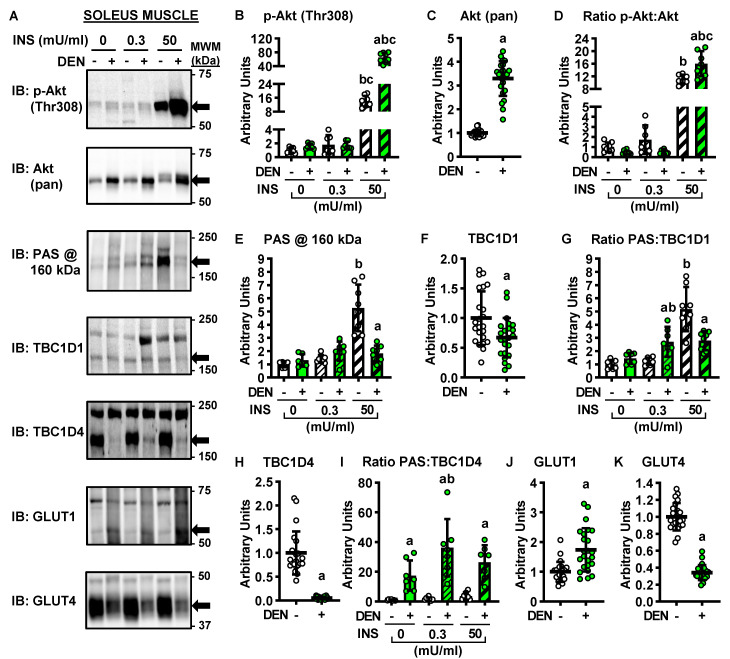
Effects of long-term denervation (DEN) on insulin signaling in the soleus muscle. In male wild-type mice, hindlimb muscle denervation was induced in one leg for 28 days while the contralateral leg was sham-operated. Soleus muscles were excised and incubated in 0, 0.3 or 50 mU/ml insulin (INS). (**A**) Representative immunoblots. (**B**–**K**) Immunoblot band quantification. Statistical significance was defined as *p* < 0.05, determined using paired *t*-tests (for pooled total proteins; panels (**C**,**F**,**H**,**J** and **K**); *n* = 22–23 muscles/group), or two-way ANOVAs and Tukey’s post-hoc analysis (panels (**B**,**D**,**E**,**G** and **I**); *n* = 6–8 muscles/group), and denoted by ‘a’ vs. -, ‘b’ vs. 0 mU/ml INS, and ‘c’ vs. 0.3 mU/ml INS. GLUT = glucose transporter; PAS = phospho-Akt substrate.

**Figure 4:**
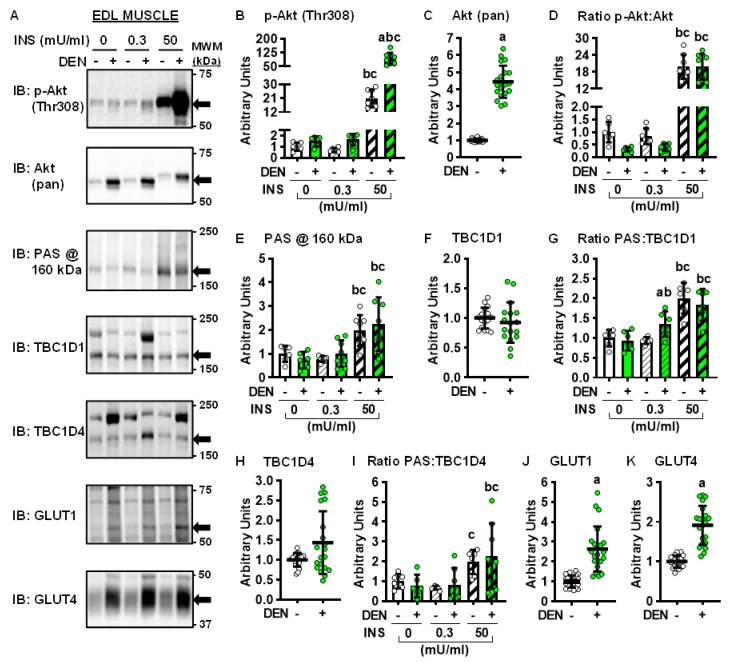
Effects of long-term denervation (DEN) on insulin signaling in the extensor digitorum longus (EDL) muscle. In male wild-type mice, hindlimb muscle DEN was induced in one leg for 28 days while the contralateral leg was sham-operated (-). EDL muscles were excised and incubated in 0, 0.3 or 50 mU/ml insulin (INS). (**A**) Representative immunoblots. (**B**–**K**) Immunoblot band quantification. Statistical significance was defined as *p* < 0.05, determined using paired *t*-tests (for pooled total proteins; panels (**C**,**F**,**H**,**J** and **K**); *n* = 17–21 muscles/group), or two-way ANOVAs and Tukey’s post-hoc analysis (panels (**B**,**D**,**E**,**G** and **I**); *n* = 6–8 muscles/group), and denoted by ‘a’ vs. -, ‘b’ vs. 0 mU/ml INS, and ‘c’ vs. 0.3 mU/ml INS. GLUT = glucose transporter; PAS = phospho-Akt substrate.

Insulin stimulates muscle glucose uptake via a signaling cascade that involves increases in the phosphorylation of Akt on Thr308, as well as phosphorylation of TBC1D1 and TBC1D4 on phospho-Akt substrate (PAS) motif sites (for review, see reference [28]). To determine whether the denervation-induced changes in muscle glucose uptake were caused by alterations in insulin signaling, immunoblot analyses were performed in soleus (Figure 3A–K) and EDL muscles (Figure 4A–K). 

In both the soleus and EDL muscles, long-term denervation did not alter Akt (Thr308) phosphorylation in the basal state or in response to submaximal insulin, but it did enhance Akt phosphorylation ~350% in response to maximal insulin (Figure 3B and Figure 4B). Denervation increased Akt protein levels ~230% in the soleus (Figure 3C) and 350% in the EDL (Figure 4C). When the ratio of Akt phosphorylation to Akt expression (i.e., phosphorylation status) was examined, this demonstrated that denervation caused an ~45% enhancement in the maximal insulin-stimulated phosphorylation status of Akt in the soleus (Figure 3D) but no change in the EDL (Figure 4D). 

In both the soleus and EDL, denervation did not alter PAS phosphorylation at 160 kDa in the basal state or in response to submaximal insulin (Figure 3E and Figure 4E). In contrast, denervation impaired maximal insulin-stimulated PAS phosphorylation at 160 kDa ~65% in the soleus but did not alter it in the EDL. Denervation decreased TBC1D1 levels ~30% and TBC1D4 levels ~95% in the soleus (Figure 3F,H) but did not significantly alter either TBC1D1 or TBC1D4 levels in the EDL (Figure 4F,H). Thus, when PAS phosphorylation status was examined, it demonstrated the following results: (1) inconsistent changes in PAS:TBC1D1 in both the soleus and EDL (Figure 3G and Figure 4G); (2) consistent denervation-induced increases in PAS:TBC1D4 in the soleus (Figure 3I); (3) no effect of denervation on PAS:TBC1D4 in the EDL (Figure 4I). Since increased signaling via Akt, TBC1D1 and TBC1D4 is associated with higher rates of muscle glucose uptake [29,30,31], collectively these findings suggest that changes in insulin signaling down to TBC1D1/TBC1D4 do not underlie the opposite effects of long-term denervation on glucose uptake between the soleus and EDL muscles.

### 2.4. Long-Term Denervation and Muscle Glucose Transporters

In skeletal muscle, basal glucose uptake is thought to be solely mediated by glucose transporter 1 (GLUT1), while insulin-stimulated glucose uptake is mediated by glucose transporter 4 (GLUT4) [32]. To determine whether the denervation-induced changes in muscle glucose uptake were caused by alterations in GLUT protein levels, immunoblots were performed. Denervation increased GLUT1 protein levels ~70% in the soleus (Figure 3J) and ~140% in the EDL (Figure 4J). In contrast, while denervation decreased GLUT4 levels ~65% in the soleus (Figure 3K), it increased GLUT4 levels ~90% in the EDL (Figure 4K). Collectively, these results suggest that changes in GLUT4 levels may be a key factor driving the sustained effects of denervation on muscle insulin sensitivity. 

### 2.5. Long-term denervation and muscle-specific GLUT4 knockout mice

**Figure 5:**
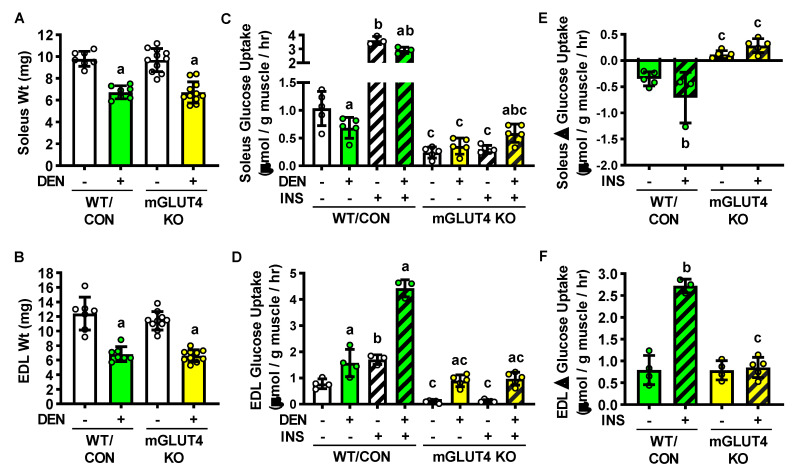
Loss of GLUT4 alters the effects of long-term denervation (DEN) on muscle glucose uptake. In male wild-type/control (WT/CON) and muscle-specific GLUT4 knockout (mGLUT4 KO) mice, hindlimb muscle DEN was induced in one leg for 28 days while the contralateral leg was sham-operated (-). (**A**) Soleus and (**B**) EDL muscle weights. (**C**,**D**) Ex vivo muscle [^3^H]-2-deoxy-D-glucose uptake was assessed in the presence of 0 or 50 mU/ml insulin (INS). (**E**,**F**) DEN-induced change in muscle glucose uptake relative to the contralateral control muscle. Statistical significance was defined as *p* < 0.05 and determined using two-way ANOVAs and Tukey’s post-hoc analysis (Panels **A**,**B**: *n* = 7–11 muscles/group; Panels **E**,**F**: *n* = 3–5 muscles/group), three-way ANOVAs, Two-Way ANOVAs and Tukey’s post-hoc analysis (**C**,**D**: *n* = 3–5 muscles/group). Significance is denoted by ‘a’ vs. -, ‘b’ vs. 0 mU/ml INS, and ‘c’ vs. WT/CON.

To directly assess the contribution of GLUT4 to the long-term denervation-induced changes in muscle glucose uptake, muscle-specific GLUT4 knockout (mGLUT4 KO) mice were examined. In both the soleus and EDL, loss of GLUT4 did not affect denervation-induced atrophy (Figure 5A,B). In both the soleus and EDL, loss of GLUT4 decreased basal and maximal insulin-stimulated glucose uptake compared to wild-type/controls (Figure 5C–F). However intriguingly in the EDL, the loss of GLUT4 did not completely prevent the denervation-induced increase in both basal and insulin-stimulated glucose uptake (Figure 5D,F). Similar results were observed in female mGLUT4 KO mice (Supplemental Figure S1). Collectively these findings suggest the following conclusions: (1) in the soleus, denervation results in sustained insulin resistance due to prolonged decreases in GLUT4 expression; (2) in the EDL, sustained denervation leads to a reversal of the initial insulin resistance due to an increase in GLUT4 protein levels. 

## 3. Discussion

The major findings from this study demonstrate that while long-term (28 days) denervation results in sustained insulin resistance in oxidative soleus muscles, it results in enhanced insulin sensitivity in glycolytic EDL muscles. In addition, this study found that these divergent denervation-induced effects were not caused by differences in the amount of muscle atrophy; signaling via Akt, TBC1D1 or TBC1D4; or changes in GLUT1 protein levels. Instead, the results indicate that alterations in GLUT4 expression play a key role in the insulin-stimulated muscle glucose uptake adaptations. 

The rapid impairment in insulin-stimulated glucose uptake following 1–3 days of denervation is associated with decreases in GLUT4 expression in both oxidative and glycolytic muscles [8,9,10,11,12,13,21,22,23,24]. Strikingly, this study demonstrated that long-term denervation results in a large (~90%) increase in GLUT4 protein levels in the glycolytic EDL muscle (Figure 4K), and that the denervation-stimulated increase in insulin-stimulated EDL muscle glucose uptake was completely impaired in the mGLUT4 KO mice (Figure 5D,E). However, surprisingly the loss of GLUT4 did not prevent the denervation-stimulated increase in basal muscle glucose uptake. Despite the denervation-stimulated increase in GLUT1 protein levels, it is unlikely that GLUT1 is the glucose transporter responsible for this effect. This interpretation is based on the data demonstrating that long-term denervation increased GLUT1 protein levels ~70% in the soleus and ~140% in the EDL (Figure 3J and Figure 4J), but that basal glucose uptake was only increased in the EDL muscle (Figure 1D). Thus, these results suggest that in response to denervation either GLUT1 is not completely localized on the cell surface and/or that its transport activity is controlled via one or more post-translational modifications. Recent work in L6 skeletal muscle myoblasts has shown that GLUT1 cell surface localization and glucose transport activity is increased by mutation of GLUT1 Ser490 to Asp490 [33], suggesting that in skeletal muscle GLUT1-dependent glucose uptake is regulated by phosphorylation. Future studies will need to determine if long-term denervation alters GLUT1 phosphorylation levels, and, if so, determine whether this phosphorylation is critical for its glucose transport activity. In addition, it is possible that another glucose transporter isoform may also be upregulated in EDL muscle in response to denervation. Our recent work has demonstrated an increase in GLUT3, GLUT6 and GLUT10 following functional overload-induced muscle hypertrophy in plantaris muscles from both wild-type/control and mGLUT4 KO mice [34], suggesting a possible role for these GLUTs in the regulation of skeletal muscle size and metabolic remodeling. Future studies will also need to determine if long-term denervation alters the expression of any of these GLUT isoforms to assess whether they could be involved in the opposing effects of denervation on muscle glucose uptake.

The rapid impairment in insulin-stimulated glucose uptake following 1–3 days of denervation has also been associated with decreases in insulin signaling via Akt [15,20]. Importantly, the results from this study demonstrated no significant impairment in basal or insulin-stimulated Akt phosphorylation status following long-term denervation in either muscle (Figure 3B and Figure 4B). These findings were largely consistent with the data showing variable effects of long-term denervation on TBC1D1 and TBC1D4 PAS phosphorylation status (Figure 3G,I and Figure 4G,I) and collectively suggest that denervation does not result in a sustained impairment in insulin signaling. However, since both TBC1D1 and TBC1D4 contain numerous phosphorylation sites and are subject to regulation by many upstream kinases (for review, see references [28,35]), additional studies are needed to fully assess the possible contribution of these proteins to the long-term denervation-induced changes in muscle glucose uptake. These future studies should include an examination of TBC1D1 and TBC1D4 phosphorylation on specific sites (e.g., TBC1D4 Thr642) as well as TBC1D1 and TBC1D4 Rab-GTPase activity. Unfortunately, due to the small size of the muscles following 28 days of denervation, we were unable to conduct these studies in these muscle samples.

Previous work in mice has demonstrated that denervation-induced muscle atrophy stabilizes 25–30 days post-injury with an ~30–55% loss of muscle weight and cross-sectional area [19,36]. Consistent with those findings, in this study, 28 days of denervation induced an ~30–40% decrease in soleus and EDL muscle weights from the male and female, wild-type, control and muscle-specific GLUT4 KO mice (Figure 1 and Figure 5 and Supplemental Figure S1). The lack of a difference in muscle atrophy response between the wild-type/control and mGLUT4 KO mice was initially surprising since muscle-specific loss of GLUT4 led to a slight (~10%), but non-significant, decrease in body weight in male mice (Figure 5A) and an ~20% decrease in body weight in female mice (Supplemental Figure S1A), suggesting a connection between muscle glucose uptake and the regulation of muscle mass. However, it is consistent with our previous work demonstrating that muscle GLUT4 expression is not required for overload-induced muscle hypertrophic growth [34]. Thus, taken together these results demonstrate that long-term denervation induces muscle atrophy independent of differences in muscle metabolic/fiber-type, gender, and GLUT4 expression.

## 4. Materials and Methods

### 4.1. Animals

Experiments were performed in accordance with the East Carolina University Institutional Animal Care and Use Committee (protocols: P066a approved on 28 July 2014 and P066b approved on 19 May 2017) and the National Institutes of Health Guidelines for the Care and Use of Laboratory Animals. Male CD-1 mice (6–7 weeks old) were obtained from Charles River Laboratories (Raleigh, NC, USA). GLUT4 LoxP mice were generated as described [37]. GLUT4 loxP mice (mixed FVB, 129, C57BL/6 background strain) were bred with muscle creatine kinase Cre recombinase transgenic mice (MCK-Cre^+^; mixed C57BL/6N and C57BL/6J background strain; The Jackson Laboratory, Bar Harbor, ME, USA) to generate the following: wild-type (WT), GLUT4 loxP^+/-^ and loxP^+/+^ mice (control), MCK-Cre^+^ (control), and muscle-specific GLUT4 knockout (mGLUT4 KO) mice. For these studies both male and female, mGLUT4 KO mice and their wild-type and control littermates (11–12 weeks old) were utilized. All mice were housed in cages at 21–22 °C with a 12 hr light/dark cycle. A standard chow diet (Prolab^®^RMH3000, cat#5P75*, PMI Nutritional International, St. Louis, MO, USA) and water were available ad libitum.

### 4.2. Unilateral Denervation Surgery

Hindlimb muscle denervation was induced by the unilateral resection of a segment of the sciatic nerve using methods previously described [26]. Briefly, mice were anesthetized with isoflurane gas (2–3%) and an ~10 mm incision made mid-thigh. Following blunt dissection, the sciatic nerve was visualized and an ~5 mm segment removed. A sham surgery was performed on the contralateral leg. Mice recovered for 28 days prior to muscle collection. After 28 days, mice were fasted overnight, anesthetized with pentobarbital sodium (90–100 mg/kg body weight) for 40 min and euthanized by cervical dislocation. Muscles were excised and weighed to ±0.1 mg using an analytical balance (model XS105, Mettler-Toledo, Columbus, OH, USA), prior to additional experimental perturbations as described below. 

### 4.3. Muscle [^3^H]-2-Deoxyglucose Uptake

[^3^H]-2-Deoxyglucose uptake was assessed in isolated muscles using methods previously described by our lab [24,32]. For insulin-stimulated glucose uptake, muscles were incubated in continuously gassed (95% O_2_, 5% CO_2_), 37 °C Krebs-Ringer-Bicarbonate (KRB) buffer containing: 117 mM NaCl, 4.7 mM KCl, 2.5 mM CaCl_2_, 1.2 mM KH_2_PO_4_, 1.2 mM MgSO_4_, and 24.6 mM NaHCO_3_ supplemented with 2 mM pyruvate for 60 min, prior to the addition of insulin (0.3 or 50 mU/ml; cat#11376497001, Sigma-Aldrich, St. Louis, MO, USA) for 20 min. To assess glucose uptake, muscles were incubated in continuously gassed, 30 °C KRB buffer supplemented with 1.5 μCi/ml [^3^H]-2-deoxy-d-glucose (cat#NET54900, Perkin Elmer, Waltham, MA, USA), 1 mM 2-deoxy-D-glucose, 0.45 μCi/ml [^14^C]-mannitol (cat#NEC314, Perkin Elmer, Waltham, MA, USA), and 7 mM mannitol for 10 min. Muscles were frozen in liquid nitrogen. Frozen muscles were weighed, solubilized in 1.0 N NaOH at 80 °C for 15 min, and the solution neutralized with 1.0 N HCl. Aliquots were removed for liquid scintillation counting of the [^3^H] and [^14^C] labels, and the extracellular and intracellular spaces calculated to determine [^3^H]-2-deoxyglucose uptake.

### 4.4. Immunoblot Analyses

To assess myosin heavy chain isoforms, muscles were homogenized in buffer containing: 100 mM Tris-HCl pH 7.5, 40 mM dithiothreitol, 5 mM EDTA, 0.0015 mM aprotinin, 3 mM benzamidine, 0.01 mM leupeptin, 1 mM phenylmethylsulfonylfluoride, and 10% sodium dodecyl sulfate. Samples were rotated end-over-end at 21–22 °C for 60 min and centrifuged at 13,000× *g* for 30 min.

To assess insulin signaling and glucose transporter levels, muscles were pre-incubated in continuously gassed 37°C Krebs buffer supplemented with 2 mM pyruvate for 60 min, prior to the addition of insulin (0.3 or 50 mU/ml) for 20 min. Muscles were frozen in liquid nitrogen. Frozen muscles were homogenized in buffer containing: 20 mM Tris-HCl pH 7.5, 5 mM EDTA, 10 mM Na_4_P_2_O_7_, 100 mM NaF, 2 mM NaVO_4_, 0.0015 mM aprotinin, 0.01 mM leupeptin, 3 mM benzamidine, 1 mM phenylmethylsulfonylfluoride, and 1% IGEPAL® CA-630. Samples were rotated end-over-end at 4 °C for 60 min and centrifuged at 14,000× *g* for 30 min. Lysate protein concentrations were determined via the Bradford method using protein assay dye (cat#5000006, Bio-Rad Laboratories, Hercules, CA, USA). Immunoblot analyses were performed using standard methods as described by our laboratory [34,38,39]. Lysates were subjected to SDS-PAGE, and proteins transferred onto nitrocellulose membranes. MemCode Reversible Protein stain (cat#PI24580, Fisher Scientific, Waltham, MA, USA) or Ponceau S solution (cat#P7170, Sigma-Aldrich, St. Louis, MO, USA) was utilized to assess equal protein loading and transfer. Immunoblotting conditions were as described in Table 1. Densitometric analysis of immunoblots was performed using a ChemiDoc XRS+ imager and Image Lab™ software (BioRad Laboratories, Hercules, CA, USA).

Antibodies and blotting conditions utilized in the immunoblotting analyses. All bovine serum albumin (BSA) and non-fat dry milk solutions were made by dissolving the reagent in a 1x Tris-buffered saline (TBS)+0.1% Tween-20 solution. Information on the primary antibodies utilized including dilution, solution diluted in, company name or developer, catalog number, lot number, and associated Research Resource Identifiers (RRIDs) are indicated in the table. The species and dilution factor for the horseradish peroxidase (HRP)-conjugated secondary antibodies are indicated in the table. All HRP-conjugated secondary antibodies were diluted in a 5% BSA, 1× TBS+0.1% Tween-20 solution. The mouse-HRP antibody was cat#12-349, lot#1785864 and lot#2365945 from Millipore (Burlington, MA, USA). The rabbit-HRP antibody was cat#PI31460, lot# QG221919 and SH253595 from Fisher Scientific (Waltham, MA, USA). The enhanced chemiluminescence (ECL) substrate reagents utilized were either the Western Lightning™ Plus-ECL, cat# NEL105001EA from Perkin Elmer Life Sciences (Waltham, MA, USA), or the Super Signal™ West Femto Chemiluminescent Substrate, cat# PI34096, from Thermo Fisher Scientific (Waltham, MA, USA). GLUT = glucose transporter; MHC = myosin heavy chain.

### 4.5. Statistical Analyses

Data sets were screened once for outliers defined as data points whose value exceeded the mean ± 2× standard deviation for that treatment group. Any identified outliers were removed prior to graphing or assessment of statistical significance. Data were graphed using GraphPad Prism v8 software (GraphPad Software Inc., San Diego, CA, USA) and are presented as scatter plots and include the mean ± standard deviation. Statistical significance was defined as *p* < 0.05 and assessed using SigmaPlot v12 software (Systat Software Inc., San Jose, CA, USA). Tests performed are indicated in the figure legends and included: paired *t*-tests, one-way ANOVA, two-way ANOVA, and/or three-way ANOVA, and Tukey’s post hoc analysis. The number of mice or muscles utilized to determine significance is indicated in the figure legends.

## 5. Conclusions

In conclusion, this study demonstrates that long-term denervation results in sustained insulin resistance in oxidative skeletal muscles but enhanced insulin sensitivity in glycolytic muscles. It further shows that these opposing effects on insulin-stimulated muscle glucose uptake are due to the protein levels of glucose transporter 4 (GLUT4). Future studies are focused on elucidating the mechanism(s) underlying the denervation-induced changes in GLUT4 expression as they could lead to novel insights towards preventing and/or treating skeletal muscle insulin resistance.

## Figures and Tables

**Table 1:** Immunoblotting conditions.

Antigen	Blocking	1° Antibody	1° Antibody RRID	2° Antibody	ECL Reagent
Akt (11E7)	5% BSA	1:2000 in 5% BSA, cat#4685, lot#6, Cell Signaling Technology, Danvers, MA, USA	AB_2225340	1:2000 Rabbit-HRP	Western Lightning™
p-Akt (Thr308)	5% BSA	1:2000 in 5% BSA, cat#9275, lot#20, Cell Signaling Technology, Danvers, MA, USA	AB_329828	1:2000 Rabbit-HRP	Western Lightning™
p-Akt Substrate (PAS)	5% BSA	1:2000 in 5% BSA, cat#9611, lot#11, Cell Signaling Technology, Danvers, MA, USA	AB_330302	1:2000 Rabbit-HRP	Western Lightning™
GLUT1	5% milk	1:4000 in 5% BSA, cat#07-1401, lot#2956779, Millipore, St. Louis, MO, USA	AB_1587074	1:2000 Rabbit-HRP	Western Lightning™
GLUT4	5% BSA	1:2000 in 5% BSA, cat#07-1404, lot#2890837 and 2987632, Millipore, St. Louis, MO, USA	AB_1587080	1:2000 Rabbit-HRP	Western Lightning™
MHC Type I	5% BSA	1:500 in 5% BSA, cat#BA-F8, lot# N/A,Developmental Studies Hybridoma Bank, University of Iowa, Iowa City, IA, USA	AB_10572253	1:5000 Mouse-HRP	EDL: Western Lightning™ Soleus: Super Signal™
MHC Type IIA	5% milk	1:500 in 5% BSA, cat#SC-71-S, lot# N/A, Developmental Studies Hybridoma Bank, University of Iowa, Iowa City, IA, USA	AB_2147165	1:5000 Mouse-HRP	Western Lightning™
MHC Type IIB	5% BSA	1:100 in 5% BSA, cat#BF-F3, lot# N/A, Developmental Studies Hybridoma Bank, University of Iowa, Iowa City, IA, USA	AB_2266724	1:5000 Mouse-HRP	EDL: Super Signal™ Soleus: Western Lightning™
MHC Type IIX	5% BSA	1:500 in 5% BSA, cat#6H1, lot# N/A, Developmental Studies Hybridoma Bank, University of Iowa, Iowa City, IA, USA	AB_1157897	1:5000 Mouse-HRP	Western Lightning™
TBC1D1 (V796)	5% BSA	1:2000 in 5% BSA, cat#4629, lot#1, Cell Signaling Technology, Danvers, MA, USA	AB_1904162	1:2000 Rabbit-HRP	Super Signal™
TBC1D4	5% BSA	1:1000 in 5% BSA, cat#07-741, lot#1962662, Millipore, St. Louis, MO, USA	AB_492639	1:2000 Rabbit-HRP	Western Lightning™

## Supplementary Materials

The following are available online at https://www.mdpi.com/article/10.3390/ijms22094913/s1, Supplemental Figure S1. Loss of GLUT4 expression differentially alters the effects of long-term denervation on skeletal muscle glucose uptake. In female wild-type/control (WT/CON) and muscle-specific GLUT4 knockout (mGLUT4 KO) mice hindlimb muscle denervation (DEN+) was induced in one leg for 28 days while the contralateral leg was sham-operated (DEN-). (A) Soleus muscle weights. (B) Soleus muscle ex vivo [^3^H]-2-deoxy-D-glucose uptake in the absence of insulin (INS-) or in the presence of 50 mU/ml insulin (INS+). (C) DEN-induced change in soleus muscle glucose uptake rates relative to the contralateral control muscle. (D) Extensor digitorum longus (EDL) muscle weights. (E) EDL muscle ex vivo [^3^H]-2-deoxy-D-glucose uptake with INS or INS+. (F) DEN-induced change in EDL muscle glucose uptake rates relative to the contralateral control muscle. Statistical significance was defined as *P* < 0.05, determined using Three-Way ANOVAs and/or Two-Way ANOVAs and Tukey’s post-hoc analysis, and denoted by ‘a’ vs. Sham, ‘b’ vs. INS-, and ‘c’ vs. WT/CON. (Panels A & D: N = 12–15 muscles/group; Panels B, C, E &F: N = 6–9 muscles/group).

## Abbreviations

DEN	Denervation
EDL	Extensor digitorum longus
GLUT	Glucose transporter
INS	Insulin
KRB	Krebs-Ringer Bicarbonate
mGLUT4 KO	Muscle-specific glucose transporter 4 knockout
MHC	Myosin heavy chain
PAS	Phospho-Akt substrate
SDS-PAGE	Sodium dodecyl sulfate-polyacrylamide gel electrophoresis
WT/CON	Wild-type/control
