# Supplementary material for: Insulin Resistance Is Not Sustained Following Denervation in Glycolytic Skeletal Muscle
Source: Int J Mol Sci. 2021 May 6;22(9):4913. doi: 10.3390/ijms22094913 (PMC8125496; doi:10.3390/ijms22094913)
Supplement: Supplementary file 1 [file ijms-22-04913-s001.zip › ijms-1209424-SI.pdf]

McMillin SL et al, *IJMS* – Supplemental Figure 1

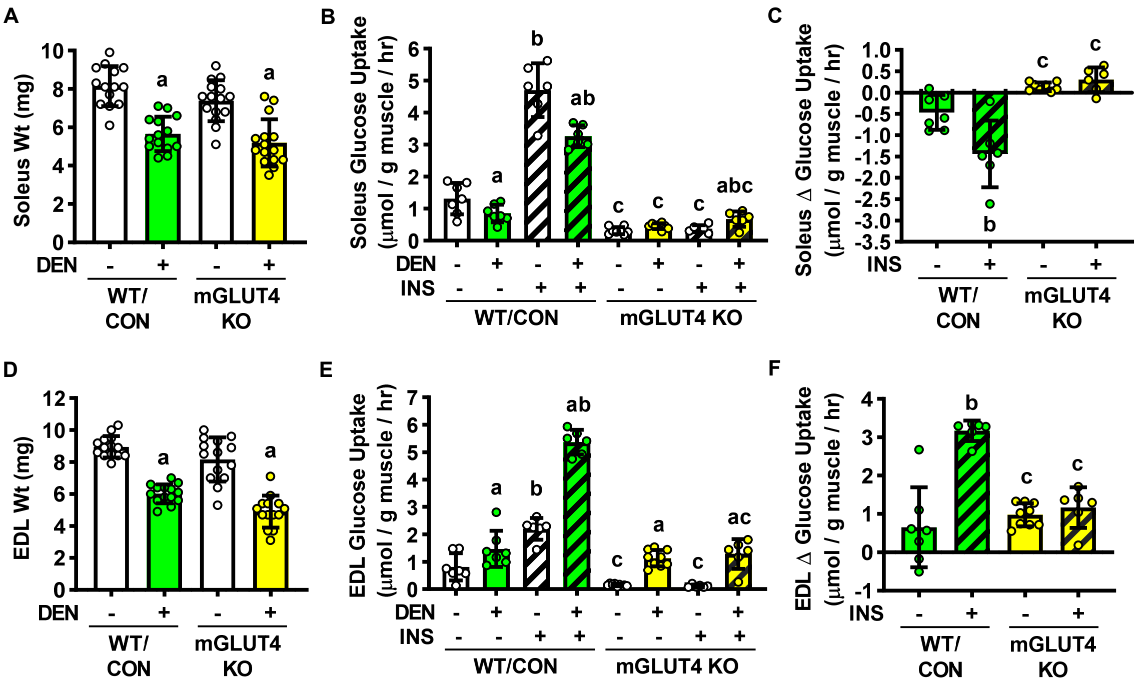

<u>Supplemental Figure S1</u>. **Loss of GLUT4 expression differentially alters the effects of long-term denervation on skeletal muscle glucose uptake.** In <u>female</u> wild-type/control (WT/CON) and muscle-specific GLUT4 knockout (mGLUT4 KO) mice hindlimb muscle denervation (DEN+) was induced in one leg for 28 days while the contralateral leg was sham-operated (DEN-). (**A**) Soleus muscle weights. (**B**) Soleus muscle ex vivo [^{3}H]-2-deoxy-D-glucose uptake in the absence of insulin (INS-) or in the presence of 50 mU/ml insulin (INS+). (**C**) DEN-induced change in soleus muscle glucose uptake rates relative to the contralateral control muscle. (**D**) Extensor digitorum longus (EDL) muscle weights. (**E**) EDL muscle ex vivo [^{3}H]-2-deoxy-D-glucose uptake with INS-or INS+. (**F**) DEN-induced change in EDL muscle glucose uptake rates relative to the contralateral control muscle. Statistical significance was defined as P<0.05, determined using Three-Way ANOVAs and/or Two-Way ANOVAs and Tukey's post-hoc analysis, and denoted by 'a' vs Sham, 'b' vs INS-, and 'c' vs WT/CON. (Panels A & D: N=12-15 muscles/group; Panels B, C, E & F: N=6-9 muscles/group)
